# Pomegranate (*Punica granatum* L.) Peel Extracts as Antimicrobial and Antioxidant Additives Used in Alfalfa Sprouts

**DOI:** 10.3390/foods11172588

**Published:** 2022-08-26

**Authors:** Manuel Reynaldo Cruz-Valenzuela, Rosa E. Ayala-Soto, Jesus Fernando Ayala-Zavala, Brenda A. Espinoza-Silva, Gustavo A. González-Aguilar, Olga Martín-Belloso, Robert Soliva-Fortuny, Filomena Nazzaro, Florinda Fratianni, Melvin R. Tapia-Rodríguez, Ariadna Thalia Bernal-Mercado

**Affiliations:** 1 Centro de Investigación en Alimentación y Desarrollo, A.C, Carretera Gustavo Enrique Astiazarán Rosas, No. 46, Col. La Victoria, Hermosillo 83304, Mexico; 2 Department of Food Technology, Agrotecnio Center, University of Lleida, Av. Rovira Roure 191, 25198 Lleida, Spain; 3 Istituto di Scienze dell’Alimentazione, ISA-CN R, Via Roma, 64, 83100 Avellino, Italy; 4 Departamento de Biotecnologia y Ciencias Alimentarias, Instituto Tecnológico de Sonora, 5 de Febrero 818 sur, Col. Centro, Ciudad Obregón 85000, Mexico; 5 Departamento de Investigación y Posgrado en Alimentos, Universidad de Sonora, Blvd. Luis Encinas y Rosales S/N, Col. Centro, Hermosillo 83000, Mexico

**Keywords:** food byproduct, phenolic compounds, anti-quorum sensing agents, natural food additive, added value

## Abstract

Aqueous and ethanolic pomegranate peel extracts (PPE) were studied as a source of phenolic compounds with antimicrobial, anti-quorum sensing, and antioxidant properties. The aqueous extract showed higher total phenolic and flavonoid content (153.43 mg GAE/g and 45.74, respectively) and antioxidant capacity (DPPH radical inhibition: 86.12%, ABTS radical scavenging capacity: 958.21 mg TE/dw) compared to the ethanolic extract. The main phenolic compounds identified by UPLC-DAD were chlorogenic and gallic acids. The aqueous PPE extract showed antimicrobial activity against *Listeria monocytogenes*, *Salmonella* Typhimurium, *Candida tropicalis* (MICs 19–30 mg/mL), and anti-quorum sensing activity expressed as inhibition of *Chromobacterium violaceum* violacein production (%). The aqueous PPE extracts at 25 mg/mL applied on alfalfa sprouts reduced psychrophilic bacteria (1.12 Log CFU/100 g) and total coliforms (1.23 Log CFU/100 g) and increased the antioxidant capacity of the treated sprouts (55.13 µmol TE/100 g (DPPH) and 126.56 µmol TE/100 g (ABTS)) compared to untreated alfalfa. This study emphasizes PPE’s antioxidant and antimicrobial activities in alfalfa sprouts preservation.

## 1. Introduction

Alfalfa sprouts possess beneficial nutritional properties as they are a good source of fiber, vitamins, minerals, and phytochemicals and have low amounts of saturated fats and sodium [1]. These properties have made alfalfa sprouts popular and highly consumed. However, they are also very susceptible to microbial contamination. Contamination of alfalfa sprouts can occur mainly during germination since nutrients, humidity, temperature, and light intensity during this process favor microbial growth [2]. Several foodborne outbreaks are linked with the consumption of sprouts, mainly caused by *Salmonella* and *Listeria monocytogenes* [3,4]. Hence, disinfection techniques must be improved by identifying alternatives to ensure the safety of these sprouts. Sodium hypochlorite (NaClO) is the most common practice used for postharvest disinfection of fresh fruits and vegetables [5]. However, its use generates secondary products, such as chloramines and trihalomethanes, which are harmful to humans [6,7]. Consumers have also expressed their interest in using natural sources as food additives to reduce the risk of consuming synthetic antioxidants and antimicrobial substances [8].

Fruit byproducts are rich sources of bioactive compounds with the potential to be used as antimicrobial and antioxidant food additives [9]. Food waste is one of humanity’s most significant challenges [10]. Primarily agricultural processing generates many byproducts and wastes, contributing to 50% of total food waste [10]. These fruits and vegetable byproducts include pulp, bagasse, peels, seeds, bran, stems of fruits and vegetables discarded by damage during transportation, storage, or the processing of juices, freezing, and minimally processed products [11]. Generally, agricultural byproducts are wasted, causing an impact on the economic and social sector, and contributing to global greenhouse gas and environmental pollution [12], highlighting the importance of finding an alternative use for byproducts.

Pomegranate (*Punica granatum* L.) is a tropical and subtropical fruit with highly nutritious compounds. Pomegranate processing generates large amounts of byproducts when obtaining juice, jams, and wine, among other products [13]. Pomegranate peel contains gallotannins, ellagitannins, anthocyanins, hydroxycinnamic acids, and hydroxybenzoic acids [14,15]. Pomegranate peel extract (PPE) has antioxidant properties and antimicrobial effects against many pathogenic microorganisms [14,16,17,18,19]. Even when several studies have reported the in vitro antimicrobial and antioxidant potential of PPE, few have recently reported their efficacy in real food systems. Drinić, Mudrić, Zdunić, Bigović, Menković and Šavikin [13] reported using PPE as an antioxidant to improve pomegranate seed oil quality and oxidative stability compared to a synthetic antioxidant. Belgacem, et al. [20] also demonstrated the potential use of PPE to ensure microbial safety and reduce the bacterial load on fresh-cut apple, melon, and pear throughout seven days of storage.

The importance of this research lies in the field of the development of natural preservatives. Fresh fruits and vegetables are highly susceptible to microbial contamination, so they must be previously disinfected or use synthetic preservatives to extend their shelf life. Consumers have shown more interest in fresh, natural, less processed, and free of chemical additives products due to the growing concern about the negative impact on consumers’ health (carcinogenic, teratogenic effect, etc.). This scenario has led to numerous investigations using natural sources as food additives. This study advances knowledge and strengthens this research by showing that PPE is a potent antioxidant and antimicrobial for alfalfa sprouts. Although other studies have been conducted in this area, few have addressed the preservation of alfalfa using extracts from natural sources. As far as the authors know, the addition of PPE to alfalfa sprouts was not studied previously; therefore, this study aimed to evaluate the effect of pomegranate peel extracts as an antimicrobial and antioxidant agent to enhance the quality of alfalfa sprouts.

## 2. Materials and Methods

### 2.1. Preparation of Pomegranate Peel Extracts

Fully ripe pomegranate fruits were obtained from organic orchards in San Pedro, Hermosillo, Sonora, Mexico. They were processed by removing the peel manually, then cut into pieces (1 cm^2^) and separated to prepare two extractions (ethanolic and aqueous). Three replicates of 10 g of pomegranate peel were placed in 100 mL of ethanol: water (7:3 *v*/*v*) for the ethanolic extraction. Samples were macerated in darkness for 10 days at 25 °C, the obtained extracts were filtered, and ethanol was vacuum evaporated to dryness at 63 rpm and 45 °C. Then, dry samples were hydrolyzed by adding 10 mL of 4 M NaOH and left for 4 h in darkness. Subsequently, acid hydrolysis was carried out with 4 M HCl until reaching pH 2 [21]. The acid and alkaline hydrolytic treatments of plant materials facilitate polyphenol recovery. Then, 30 mL of the resulting dispersions were poured into 50 mL vials and freeze-dried. The aqueous extraction was carried out by weighing three replicates of 1 g of dried and crushed pomegranate peel and mixed with 40 mL of distilled water. This mixture was placed in a water bath (100 °C) for 30 min, and then it was filtered, freeze-dried, and stored at −35 °C for later use [22]. Each extract was analyzed to identify and quantify phenolic compounds, antioxidant capacity, and antimicrobial activity to select the more active treatment for its use in alfalfa sprouts.

### 2.2. Identification and Quantification of Individual Phenolic Compounds

The main phenolic compounds in the peel extracts were quantified using ultra-performance liquid chromatography. These analyses were performed using a Waters system linked simultaneously to a PDA 2996 photodiode array detector. The ultraviolet detection wavelength was set at 280 nm. The software Empower (Waters, Milford, MA, USA) was used for data acquisition and processing. The analysis was performed at 30 °C using a reverse-phase column (BEH C18, 1.7 mm, 2.1 × 100 mm, Waters). The mobile phase consisted of solvent A (7.5 mM acetic acid) and solvent B (acetonitrile) at a flow rate of 250 mL/min [23]. Three replicates of each extract were analyzed with an injection volume of five μL. The elution gradient was from 5% solvent B for 0.8 min, B 5–20% solvent for 5.2 min, 20% isocratic solvent B for 0.5 min, solvent B 20–30% for one minute, 30% solvent Isocratic B for 0.2 min, 30–50% solvent B for 2.3 min, 50–100% solvent B for one minute, 100% isocratic solvent B for one minute, and finally 100 – 5% solvent B for 0.5 min. At the end of this sequence, the column was balanced in the initial conditions for 2.5 min. The pressure ranges from 6000 to 8000 psi during the execution of the analysis. The effluent was introduced to a liquid chromatography detector at a range of 210–400 nm and 1.2 nm of resolution. Identification was made by comparing UV-Vis spectra and retention time of each sample peak with pure standards, and quantification was done by relating the area under the curve of each identified peak with standard curves; results were expressed as μg of phenolic compound per g of extract.

### 2.3. Total Phenolic and Flavonoid Content

The total phenol content of both extracts was determined according to the Folin-Ciocalteau method with some modifications [22]. In a microplate well, 75 μL of Folin reagent (1:10), 15 μL of each extract, and 60 μL of 7.5% Na_2_CO_3_ were added. These mixtures stood in the dark for 30 min, and the optical density (OD) at 748 nm was measured using a microplate reader (FLUOstar omega-BMG LABTECH). A calibration curve for gallic acid was prepared, and results were expressed as mg of gallic acid equivalents (GAE)/g of extract.

Total flavonoids were quantified by the method described by Vazquez-Armenta, Silva-Espinoza, Cruz-Valenzuela, Gonzalez-Aguilar, Nazzaro, Fratianni and Ayala-Zavala [22] with some modifications. Within 2 mL vials, 100 μL of each sample were added and mixed with 430 μL of solution A (1.8 mL of 5% NaNO_2_ with 24 mL of distilled water) and allowed to stand for 5 min. Subsequently, 30 μL of 10% AlCl_3_ were added and left to stand for 1 min. Finally, 440 μL of solution B (12 mL of 1M NaOH with 14.4 mL of distilled water) were added. From this reaction, 150 μL were poured into microplate wells to read the OD at 496 nm and compared to a standard curve of quercetin. Results were expressed as mg of quercetin equivalents (QE)/g of extract.

### 2.4. Antioxidant Capacity

For this assay, 10 μL of each extract were mixed by triplicate in microplates with 140 μL of the adjusted DPPH (2,2-diphenyl-1-picrylhydrazyl radical). The extract-radical mixture was allowed to stand for 30 min in darkness after reading its OD at 518 nm [24]. The results were expressed as percentage of DPPH inhibition. For the ABTS (2,2′-azino-bis(3-ethylbenzothiazoline-6-sulfonic acid)) assay, 19.3 mg of the ABTS radical was dissolved in 5 mL of distilled water. Separately, a solution of K_2_S_2_O_8_ (37.8 mg in 1 mL of distilled water) was prepared. Subsequently, 88 μL of this solution was added to 5 mL of ABTS solution. This mixture stood 16 h in darkness; then, 0.5 mL was added to 35 mL of pure ethanol, adjusting the OD at 754 nm to 0.7. Subsequently, three replicates of 5 μL of each extract and 245 μL of the previously prepared free radical were added to a microplate, left in darkness for 5 min, and the final OD_754_ was measured [24]. Results were expressed as mg of Trolox equivalents (TE)/g of extract.

### 2.5. In Vitro Antimicrobial Activity

The antimicrobial capacity of the pomegranate peel extracts was tested against *Listeria monocytogenes* ATCC 7644, *Salmonella enteric* subsp. enteric serovar Typhimurium ATCC 14028, *Chromobacterium violaceum* ATCC 12472, and *Candida tropicalis* ATCC 1369 using the broth microdilution technique. For this, 5 μL of microbial inoculum (6 log CFU/mL) and 295 μL of diluted extracts in Muller Hinton broth (0–200 mg/mL) were mixed and incubated for 24 h at 37 °C. The lowest concentration of the extract that caused a visual growth inhibition was taken as the minimum inhibitory concentration (MIC), and this effect was confirmed by measuring the OD at 600 nm after the incubation time [22]. The experiment was repeated three times, and the results were expressed as mg/mL.

### 2.6. Anti-Quorum Sensing Potential

The violacein production by *C. violaceum* exposed to the aqueous extract was determined in conjunction with bacterial viability to evaluate the *quorum sensing* interruption. For the assay, 1 mL of inoculum (1 × 10^6^ CFU/mL) treated with each different concentration of PPE (doses determined considering MIC values) at 25 °C for 24 h was centrifugated (15,800× *g* for 10 min) to precipitate violacein [25]. Then, the supernatant culture was removed; the obtained pellet was solubilized in 1 mL of dimethyl-sulfoxide and centrifuged (15,800× *g* for 10 min) twice to discard cells. The OD of each tube with violacein supernatant was measured in a microplate reader Fluostar Omega (BMG Labtech, Chicago, IL, USA) at 585 nm. Cell viability of the treated bacteria was registered and expressed as log CFU/mL; this assay was performed by triplicate, and results were expressed as violacein production (%).

### 2.7. Application of PPE to Alfalfa Sprouts

Fully grown alfalfa sprouts were purchased from a local producer. Sprouts were immersed for 2 min within different treatments, then were dried and stored until further analysis [26]. Treatments were carried out with the aqueous extract of pomegranate peel at two concentrations (25 mg/mL and 50 mg/mL); two controls were used: sodium hypochlorite 200 ppm and sterile water. Only the aqueous extract was evaluated as it was more effective. The physicochemical characteristics were evaluated as follows: Titratable acidity (% citric acid) and pH were determined from 10 g of sample homogenized in 50 mL of distilled water, using an automatic titrator Mettler (Model DL21); results were expressed as pH and mg of citric acid per fresh weight. Total soluble solids were measured with an Abbé digital refractometer, and results were shown as % of total soluble solids. The physicochemical characteristics of the treated alfalfa sprouts were titratable acidity 0.260% of citric acid, pH 6.11, and 5.16 °Brix of total soluble solids.

### 2.8. Microbiological Analysis of Treated Alfalfa Sprouts

Treated alfalfa sprouts (10 g) were sampled, diluted into 90 mL of peptone water, and homogenized for 10 min. Serial dilutions were carried out to determine microbial counts. Total psychrophilic bacteria were counted by incubating sample dilutions (1 mL) in plate count agar for 7 days at 4 °C. Total coliform bacteria were enumerated in red-violet bile agar after incubating at 35 °C for 24 h. Total fungi and yeasts were incubated for 5 days at 25 °C using potato dextrose agar. Once the incubation time elapsed, colonies were counted and transformed to Log_10_ of colony-forming units per gram of sample (log CFU/g).

### 2.9. Antioxidant Capacity of Treated Alfalfa Sprouts

To measure the antioxidant capacity, 10 g of each treatment’s sample was homogenized in 20 mL of 80% methanol. The homogenate was sonicated (Branson 2510R-DTH) for 30 min at 1 °C. Then, it was centrifuged (Beckman Coulter centrifuge, Allegra 64R) at 5000× *g* for 15 min at 4 °C and the supernatant was filtered through Whatman No. 1 paper. This procedure was repeated twice with 10 mL of 80% methanol; each volume was collected, filtered, and finally brought to 50 mL with methanol (80%). The extracts were evaluated for antioxidant capacity by the methods described above (DPPH and ABTS). The antioxidant capacity of the sprouts was expressed as μmol of TE/100 g [26].

### 2.10. Statistical Analysis

A completely randomized experimental design was carried out to evaluate the effect of the extraction on the bioactive properties of pomegranate extracts. The factor to be analyzed was the type of extraction (aqueous or ethanolic), and the response variables were violacein production, cell viability, and antioxidant capacity (DPPH and TEAC). A completely randomized block design was carried out to evaluate the effect of the pomegranate extract treatments on the alfalfa sprouts’ microbial load and antioxidant capacity. The analyzed factors were the extract dose and storage time, which were blocked to see the effect of the treatments on the antimicrobial and antioxidant capacity. Both experiments included an analysis of variance (ANOVA) to estimate significant differences (*p* ≤ 0.05) among treatments, and the mean comparison test was made using the Tukey-Kramer method using the NCSS program.

## 3. Results and Discussion

### 3.1. Identification and Quantification of Phenolic and Flavonoid Content and the Antioxidant Capacity of PPE

Table 1 shows the phenolic and flavonoid content of aqueous and ethanolic PPE. Even when the aqueous extract showed significantly higher phenolic content than the ethanolic extract, no significant difference (*p* > 0.05) was found in total flavonoid content. Also, Table 1 shows the antioxidant results of the DPPH and ABTS assays.

**Figure 1:**
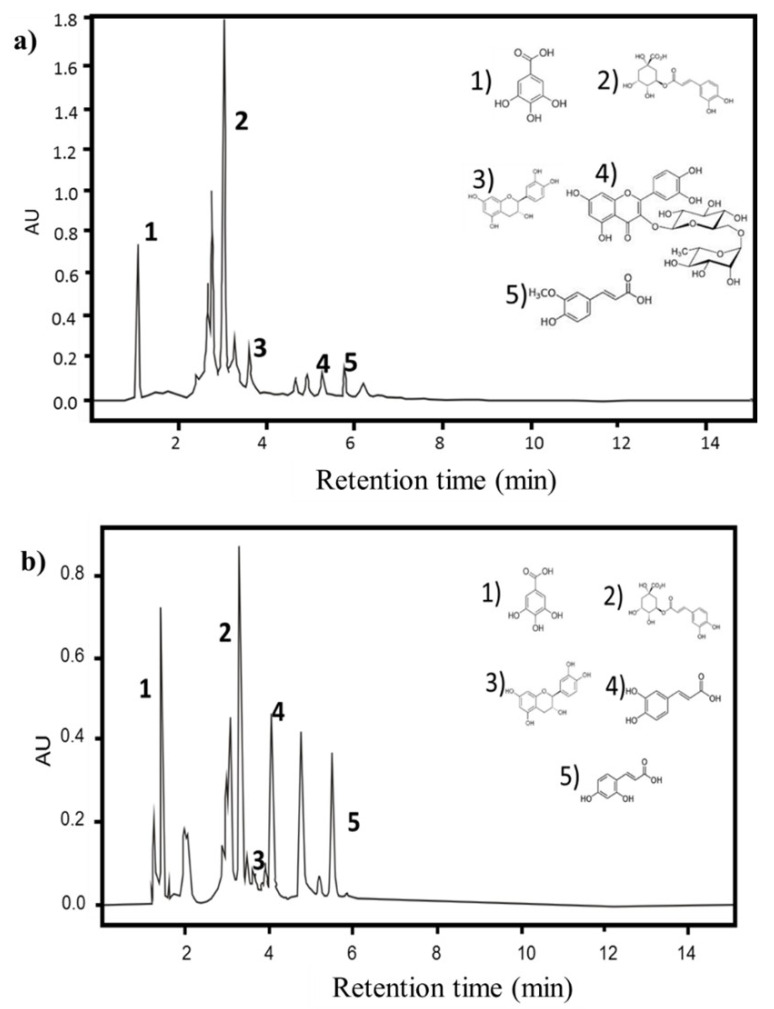
Identification of phenolic compounds of (**a**) the aqueous extract: 1. Gallic acid; 2. Chlorogenic acid; 3. Catechin; 4. Rutin and 5. Ferulic acid; and (**b**) ethanolic extract: 1. Gallic acid; 2. Chlorogenic acid, 3. Catechin; 4. Caffeic acid and 5. *p*-Cumaric acid.

Figure 1a shows the phenolic compound profile present in the aqueous PPE. Five compounds were found in this extract: gallic acid, chlorogenic acid, catechin, rutin, and ferulic acid. The main component was the chlorogenic acid with a 2990.89 μg/g dw, followed by rutin with 514.19 μg/g dw. Figure 1b shows the phenolic composition corresponding to the ethanolic extract, and five compounds were identified: chlorogenic acid, caffeic acid, gallic acid, *p*-coumaric acid, and catechin. The main components were chlorogenic acid with 1540.31 μg/g dw and caffeic acid with 399.37 μg/g dw. More phenolic compounds were found in the aqueous extract, which could explain its higher antioxidant activity.

The results presented in this study are similar to those of other authors who reported that PPE is a good source of phenolic and flavonoid compounds with antioxidant activity. Derakhshan, Ferrante, Tadi, Ansari, Heydari, Hosseini, Conti and Sadrabad [17] reported that ethanolic PPE from different varieties revealed a range of phenolic content of 276–413 mg GAE/g and a flavonoid content in a range of 36–54 mg rutin/g. These PPEs also exhibited an antioxidant capacity of 45–58%. Kharchoufi, Licciardello, Siracusa, Muratore, Hamdi and Restuccia [15] indicated that water PPE poses an antioxidant activity of 3497.02 mmol Trolox/g. Moreover, some studies have suggested differences between extraction processes. Saad, et al. [27] reported a total phenolic content of 174.5 mg GAE g for the aqueous PPE and 6.48 mg/g for the ethanolic extract. These values are similar to the present study’s results, where the aqueous extract showed higher total phenolic content than the ethanolic extract. Other studies have described that ethanol-water mixtures could be the optimal extraction process. Živković, et al. [28] reported that the optimal conditions of ultrasound-assisted extraction of polyphenolic compounds in pomegranate peel using surface methodology surface were 25 min extraction, ethanol at 59%, a solid to solvent ratio of 1:44, and a temperature of 80 °C. The type of solvent (ethanol or water), solvent ratio, particle size, temperature, and time can be adjusted to obtain the maximum level of phenolic compounds [14].

Aqueous extract showed higher antioxidant and phenolic compounds compared to ethanolic extract. Some studies have shown that heat favors the release of phenolic compounds from plant materials. For example, a study concluded that suitable heat treatment could reasonably improve the antioxidant capacity of citrus peel. The results showed that after heat treatment, the free fraction of phenolic acids increased and that phenolic compounds could be destroyed when heated at higher temperatures for a long time (for example, 120 °C for 90 min or 150 °C for 30 min) [29]. Other studies have also shown that heat can destroy phenolic compounds but has a higher antioxidant capacity. [30]. Pomegranate peel contains a significant quantity of ellagitannins such as punicalagin, characterized by good water solubility. Although it was not possible to identify them in the extract, it is possible that they are found in greater quantity in the aqueous extract and not in the ethanolic one, explaining the reason for the higher antioxidant and phenolic content.

Some factors like the solvent used to extract phenolic compounds must be considered to obtain a particular phenolic profile since the solvent can extract different quantities of compounds due to its polarity [21]. Hydrolysis with acids or bases during the extraction process can influence the profile of phenolic compounds since hydrolysis can release some bounded complex phenolics into simpler molecules [31]. This fact could be observed in the present study, and the PPE presented compounds such as chlorogenic acid and rutin instead of larger molecules like punicalagin or ellagitannins commonly found in PPE.

The phenolic composition of each part of the pomegranate fruit is influenced by the environmental conditions, cultivar, and maturity, among others. PPE has punicalagin, gallic acid, ellagic acids, ellagitannins, gallotannins, hydroxycinnamic acids, catechins, hydroxybenzoic acids, and anthocyanins [14,15]. Some studies have indicated that the major phenolic compounds in PPE are gallic acid, ellagic acid, and punicalagin derivatives [15]. It has been reported that phenolic acids, flavonoids, and tannins compounds, especially ellagitannins, in pomegranate peel are responsible for the antioxidant activity and other bioactive properties [32]. The antioxidant mechanism of these compounds is linked to the number and position of the hydroxyl groups. Phenolic acids and flavonoids can scavenge free radicals through hydrogen atom donation, thus quenching harmful species. In addition, some phenolic compounds with adjacent hydroxyl groups, such as catechol, could chelate metals that inhibit the propagation of free radicals [14,22].

Pomegranate peel extracts showed more antioxidant potential and higher phenolic compounds than other parts such as the seeds and juices [17]. Furthermore, the phenolic content of PPE is higher than those reported in other studies with different fruit byproducts such as mango peel (70.1 mg GAE/g and 21.2 mg quercetin equivalent (QE)/g) [33] and papaya peel (63.59 mg GAE/g and 23.45 mg QE/g) [34]. It was also found that byproducts, mainly the peel, had the highest antioxidant content; this may be since the function of the peel is to protect the whole fruit, which is the reason for observing a higher concentration of phenolic compounds in this tissue [35].

### 3.2. Antimicrobial Activity against Pathogenic Bacteria and Anti-Quorum Sensing Properties of PPE

Both extracts were effective against bacteria and yeast using the broth microdilution test (Table 2). The literature has widely discussed the efficacy of PPE in inhibiting or reducing the growth of a wide range of microorganisms [36,37,38]. Kharchoufi, Licciardello, Siracusa, Muratore, Hamdi and Restuccia [15] described that a water PPE at 0.361 g/mL reduced 3.15 Log CFU/mL of *Pseudomonas putida* and 2.52 Log CFU/mL of the viability of *Saccharomyces cerevisiae*. However, the results in the present study cannot be compared due to the different microorganisms evaluated. Alexandre, Silva, Santos, Silvestre, Duarte, Saraiva and Pintado [16] reported the MICs of other PPE performed by high pressure and enzymatic assisted extraction against *Staphylococcus aureus*, *Bacillus cereus*, *Escherichia coli*, *L. monocytogenes*, *Salmonella enteritidis*, and *P. aeruginosa*. Specifically, the MICs reported against *L. monocytogenes* ranged between 7.82 to 31.25 mg/mL, and for *Salmonella enteritidis,* the MIC obtained for all the extracts was 62.5 mg/mL. These results agree with the MIC of *L. monocytogenes* in the present study which is in the range reported; however, in the case of *Salmonella,* the PPE was more effective.

The MICs of both extracts against *L. monocytogenes* (Gram-positive) were lower compared to the doses needed to interrupt the growth of *S.* Typhimurium (Gram-negative) and *C. tropicalis* (fungi). This difference can be attributed to the outer lipid membrane covering the whole cell of Gram-negative bacteria, while Gram-positive bacteria only have one membrane. This double-membrane characteristic is an essential factor in the resistance of Gram-negative bacteria to the presence of antibacterial agents [39]. *S.* Typhimurium sensitivity against the aqueous extract did not show a relation with the Gram classification; the mechanism by which it is inhibited may be due to the interruption of other cellular processes. It has been suggested that fungi cells are more resistant to antimicrobial agents than bacterial cells [15].

PPE is a potent antimicrobial against many microorganisms, including Gram-negative, Gram-positive, mold, and yeast, that can cause food spoilage and food illnesses [38]. The antibacterial effect of PPE can be attributed to the abundance of phenolic compounds [18]. As was stated before, the major compound found in the aqueous PPE was chlorogenic acid. It has been reported that chlorogenic acid can interact with the bacterial outer membrane, rupture the cell membrane, deplete intracellular content, and release macromolecules from the cytoplasm leading to bacterial death [40]. Similar mechanisms have been proposed for gallic acid and ferulic acid, a similar compound of caffeic acid. These compounds can affect the cell membrane of bacteria due to a change in charge and hydrophobicity of the cell surface [41]. In general, phenolic compounds can inhibit the activity of essential proteins by interacting with the sulfhydryl groups [18]. Phenolic compounds also have antifungal activity with similar mechanisms of action. It has been suggested that chlorogenic acid affects *Candida* cells by damaging their membranes by disrupting membrane potential [42]. Similarly, caffeic acid derivatives can affect *Candida* cytoplasmatic membrane and interfere with 1,3-β-glucan synthase [43]. Some of these mechanisms can be achieved with the PPE.

**Figure 2:**
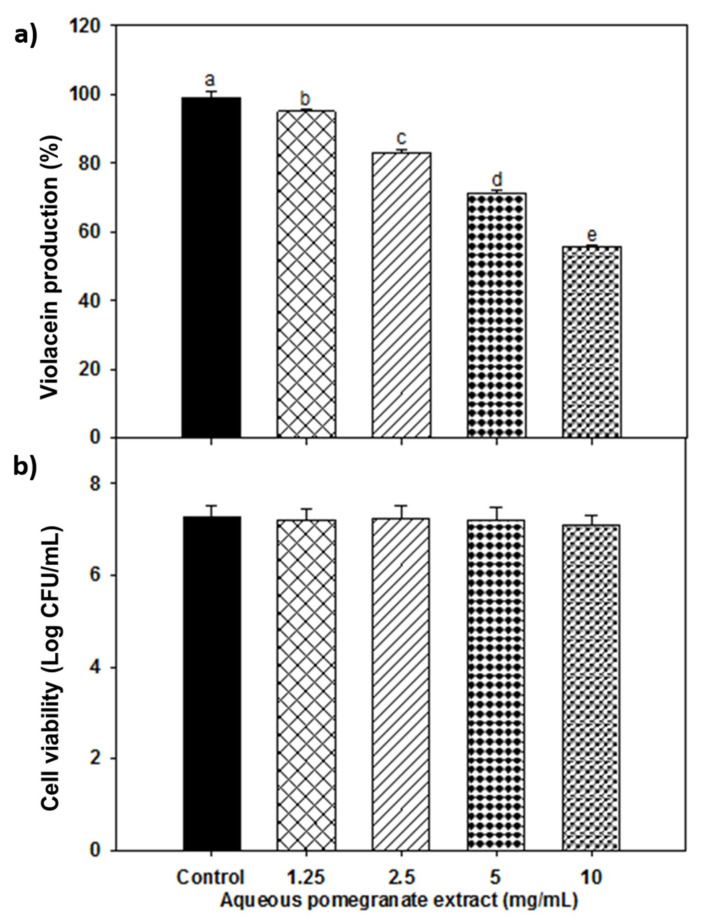
(**a**) Inhibition of violacein production of *Chromobacterium violaceum* exposed to aqueous pomegranate extract at different doses. Different letters among bars indicated significant differences (*p* ≤ 0.05). (**b**) Cell viability of *C. violaceum* after 24 h incubation with aqueous pomegranate treatments.

The anti-quorum sensing activity of aqueous PPE was also evaluated as this extract showed more efficiency against the tested biosensor bacteria. First, the MIC was determined against *C. violaceum* (Table 2). The inhibition of violacein in *C. violaceum* exposed to aqueous PPE is shown in Figure 2a. It was observed that increasing the dose of PPE higher violacein inhibition, demonstrating a maximum reduction of 43% at 10 mg/mL (*p* ≤ 0.05). Besides, cell viability (Figure 2b) showed no significant differences between control and treatments (*p* > 0.05).

The potential of PPE to inhibit *quorum sensing* communication is very promising since this system is responsible for regulating gene expression based on cell density that promotes many virulence factors of bacteria such as motility, production of toxins, sporulation, and biofilm formation, among others. In the present study, PPE decreased the percentage of violacein production, the quorum-sensing system’s product, in response to the self-production and detection of acyl-homoserine lactones in Gram-negative bacteria [25]. Few studies have reported the efficacy of PPE as a *quorum-sensing* inhibitor. Yang, Wang, Gao, Liu, Feng, Wu, Baloch, Cui and Xia [19] indicated that the tannin-rich fraction from pomegranate rind reduced violacein pigment production of *C. violaceum.* This anti-quorum activity was related to the reduction in motility and biofilm formation of *E. coli*. Similarly, punicalagin, an important component of pomegranate rind, reduced violacein production of *C. violaceum* and other virulence factors such as motility, invasion, and virulence gene expression of *Salmonella.* The results of this study suggested that PPE could be exploited as a food additive due to its antimicrobial and anti-quorum sensing activities.

### 3.3. Antioxidant Status and Microbial Counts of Alfalfa Sprouts Treated with PPE

**Figure 3:**
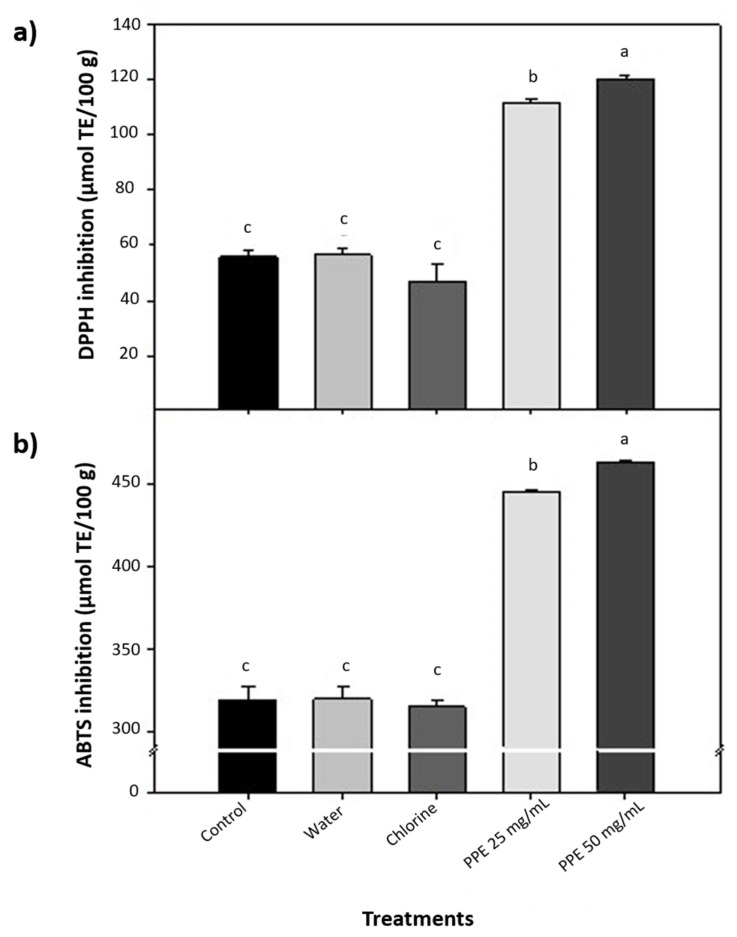
Antioxidant capacity of alfalfa sprouts treated with chlorine at 200 ppm and aqueous extract of pomegranate peel at two concentrations. (**a**) DPPH radical inhibition and (**b**) ABTS radical inhibition. Different letters among treatments mean significant differences (*p* ≤ 0.05).

The aqueous PPE showed the highest phenolic content, antioxidant capacity, and antimicrobial activity; therefore, it was selected to be applied on alfalfa sprouts. The antioxidant activity of treated alfalfa sprouts using the DPPH and ABTS assays is shown in Figure 3. The water and chlorine treatments did not increase the antioxidant activity of the alfalfa sprouts compared to the control; on the contrary, the chlorine treatment caused a slight reduction. The aqueous PPE applied at 50 mg/mL exhibited the highest antioxidant capacity determined by both assays than the rest of the treatments (*p* ≤ 0.05). The PPE at 50 mg/mL almost doubles the DPPH inhibition activity compared to control sprouts and increases 46% the ABTS radical inhibition. The PPE at 25 mg/mL also increased by 91% and 40% the antioxidant activity of alfalfa sprouts measured by DPPH and ABTS assays, respectively. These results demonstrated that applying PPE increases these vegetables’ antioxidant status.

Alfalfa sprouts are a significant source of vitamins, minerals, and phytochemicals with beneficial health properties. Few studies have reported enhancing the antioxidant status of alfalfa sprouts using plant extracts; in this sense, the present study will be the first to demonstrate the efficacy of plant byproduct extracts in increasing the antioxidant potential of alfalfa sprouts. Previous studies have described the efficacy of PPE as an antioxidant additive; for example, Drinić, Mudrić, Zdunić, Bigović, Menković and Šavikin [13] reported using PPE as an antioxidant to improve the quality and oxidative stability of pomegranate seed oil compared to a synthetic antioxidant. The 0.5% of PPE also increased the antioxidant activity of tomato and orange juice with strawberries [44], the PPE in combination with chitosan and alginate to formulate coatings maintained the quality of guavas for 20 days [45]. The chitosan-pullulan edible coating enriched with PPE maintained the antioxidant quality of green bell pepper in storage at different temperatures [46].

The results of aqueous PPE, water, and sodium hypochlorite to reduce the microbial load of alfalfa sprouts are observed in Table 3. The initial microbial load in alfalfa sprouts was 5.724 CFU/100 g for total coliforms and 5.987 CFU/100 g for psychrophiles. Fungal and yeast growth was not observed in alfalfa sprouts. All treatments significantly reduced total coliforms and psychrophiles (*p* ≤ 0.05). The aqueous PPE applied at 50 mg/mL exhibited the highest reduction (≈20–26%) of coliform and psychrophilic counts than the other treatments (*p* ≤ 0.05). The decreases in total coliform counts were 20%, 13%, and 16% for PPE at 25 mg/mL, chlorine, and water treatments, respectively, while for psychrophilic counts, the reductions were 20%, 17%, and 4%, respectively. Water treatments reduced microbial growth due to the physical removal from sprout surfaces. These results demonstrated that aqueous PPE applied in alfalfa sprouts could be used to reduce the native microflora of these vegetables. The counts of psychrophiles and coliforms were evaluated because these groups are indicators of contamination, so the reduction of these counts by treatments can ensure safety in fresh vegetable products.

The growing demand for safe and nutritious produce has promoted several disinfection technologies to reduce the microbial load of vegetables, including sprouts [47]. Similar reductions have been reported with chlorine treatments in alfalfa sprouts; for example, chlorine dioxide reduced 1.5–1.8 log CFU/g the inoculated population of *E. coli* and *S. enteritidis* [48]. The conventional method to maintain microbial safety in vegetables is through washing and disinfection with chlorine; however, it has been hypothesized that some chlorination byproducts may have carcinogenic potential. This has encouraged the quest for safe and effective alternatives [49].

As far as the authors know, the addition of PPE to alfalfa sprouts was not studied previously. Moreover, few studies have been conducted using plant extracts as disinfectants in alfalfa sprouts, despite the fact that these vegetables are very susceptible to contamination [2]. Jaroni and Ravishankar [50] demonstrated that the aqueous infusion of *Hibiscus sabdariffa* flower reduced 1 log CFU/g of the initial count of *S.* Newport after 5 min of exposure on alfalfa sprouts. After 24 h, the extract reduced the population to undetectable levels. On the other hand, some studies have reported the efficacy of PPE in reducing microbial loads in several food systems. For example, Belgacem, Schena, Teixidó, Romeo, Ballistreri and Abadias [20] demonstrated the potential use of PPE to ensure microbial safety and reduce the bacterial load on fresh-cut apple, melon, and pear throughout seven days of storage.

The maximum limits for a fresh-cut vegetable to be suitable for consumption are 5 × 10^7^ and 1 × 10^6^ CFU/100 g for total coliforms and psychrophiles, respectively [51]. In this study, alfalfa sprouts treated with aqueous PPE showed counts below these established limits. In addition, PPE increases the antioxidant potential of alfalfa sprouts; therefore, it could be an excellent natural preservative for fresh vegetables.

## 4. Conclusions

This study demonstrated that the aqueous PPE is a better source of phenolic compounds with an antioxidant activity than ethanolic extract. This extract also revealed antimicrobial activity against *L. monocytogenes* and *S.* Typhimurium (MIC = 19 mg/mL) and effectively inhibited the *quorum sensing* system of *C. violaceum* (MIC = 10 mg/mL). The aqueous pomegranate peel extract was applied to alfalfa sprouts for disinfection, resulting in a reduction in the microbial load of total coliforms (1.12–1.16 Log CFU/mL) and psychrophilic bacteria (1.23–1.58 Log CFU/mL) compared to the control. An increase (40–100%) in the antioxidant capacity of the alfalfa sprouts treated with aqueous PPE was obtained. These results showed that the pomegranate peel extract could be exploited to develop a new and safe broad-spectrum natural antimicrobial and antioxidant in foods. Future research is needed to analyze the sensory impact of pomegranate peel extract in alfalfa sprouts and its evaluation in diverse food matrices to exploit its potential as a food additive. In addition, it is necessary to complement phenolic compounds identification by gas chromatography and mass spectrophotometry (GCMS).

## Figures and Tables

**Table 1:** Phenolic compounds content and antioxidant potential of pomegranate peel extracts (PPE).

	Phenolic Content (mg GAE/g dw)	Flavonoid Content (mg QE/g dw)	DPPH Inhibition (%)	ABTS Scavenging (mg TE/g)
Aqueous PPE	154.53	45.74	86.12 ^a^	958.21 ^a^
Ethanolic PPE	111.81	50.45	76.26 ^b^	767.17 ^b^

Different letters in the same column mean significant difference between PPE extracts (*p* ≤ 0.05).

**Table 2:** In vitro antimicrobial activity of pomegranate peel extracts (PPE).

	*L. monocytogenes*	*S.* Typhimurium	*C. tropicalis*	*C. violaceum*
	Minimum Inhibitory Concentration (mg/mL)			
Aqueous PPE	19	19	30	10
Ethanolic PPE	24	24	30	26

**Table 3:** Effect of aqueous extract on total counts of coliform and psychrophilic bacteria of treated alfalfa sprouts.

	Coliform		Psychrophilic	
	Log CFU/100 g	Reduction Log	Log CFU/100 g	Reduction Log
Control	5.72 ^a^	-	5.98 ^a^	-
Water	4.95 ^b^	0.94	5.69 ^a^	0.28
Chlorine 200 ppm	4.77 ^c^	0.76	4.91 ^b^	1.07
Aqueous extract 25 mg/mL	4.60 ^d^	1.12	4.75 ^c^	1.23
Aqueous extract 50 mg/mL	4.55 ^e^	1.16	4.39 ^d^	1.58

Different literals among treatments indicate significant differences (*p* ≤ 0.05).

## Data Availability

The data that support the findings of this study are available on the repository section of the Centro de Investigacion en Alimentacion y Desarrollo (CIAD): “Extracto de cáscara de granada como antimicrobiano y potenciador antioxidante en germinados de alfalfa. Available online: http://ciad.repositorioinstitucional.mx/jspui/handle/1006/271 (accessed on 24 August 2022)”.
